# A Path towards Timely VAP Diagnosis: Proof-of-Concept Study on Pyocyanin Sensing with Cu-Mg Doped Graphene Oxide

**DOI:** 10.3390/bios14010048

**Published:** 2024-01-16

**Authors:** Mohammad Noorizadeh, Mithra Geetha, Faycal Bensaali, Nader Meskin, Kishor K. Sadasivuni, Susu M. Zughaier, Mahmoud Elgamal, Ali Ait Hssain

**Affiliations:** 1 Department of Electrical Engineering, College of Engineering, Qatar University, Doha 2713, Qatar; nader.meskin@qu.edu.qa; 2 Department of Mechanical and Industrial Engineering, Centre for Advanced Materials, Qatar University, Doha 2713, Qatar; mithra.geetha@qu.edu.qa (M.G.); kishorkumars@qu.edu.qa (K.K.S.); 3 College of Medicine, QU Health, Qatar University, Doha 2713, Qatar; szughaier@qu.edu.qa (S.M.Z.); mahmoud.elgamal@qu.edu.qa (M.E.); 4 Medical Intensive Care Unit, Hamad Medical Corporation, Doha 3050, Qatar; ahssain@hamad.qa

**Keywords:** biosensors, bio-signal processing, DNA aptamer, electrochemistry, nosocomial infections, ventilator-associated pneumonia (VAP)

## Abstract

In response to the urgent requirement for rapid, precise, and cost-effective detection in intensive care units (ICUs) for ventilated patients, as well as the need to overcome the limitations of traditional detection methods, researchers have turned their attention towards advancing novel technologies. Among these, biosensors have emerged as a reliable platform for achieving accurate and early diagnoses. In this study, we explore the possibility of using Pyocyanin analysis for early detection of pathogens in ventilator-associated pneumonia (VAP) and lower respiratory tract infections in ventilated patients. To achieve this, we developed an electrochemical sensor utilizing a graphene oxide–copper oxide-doped MgO (*GO* − *Cu* − *Mgo*) (GCM) catalyst for Pyocyanin detection. Pyocyanin is a virulence factor in the phenazine group that is produced by *Pseudomonas aeruginosa* strains, leading to infections such as pneumonia, urinary tract infections, and cystic fibrosis. We additionally investigated the use of DNA aptamers for detecting Pyocyanin as a biomarker of *Pseudomonas aeruginosa*, a common causative agent of VAP. The results of this study indicated that electrochemical detection of Pyocyanin using a GCM catalyst shows promising potential for various applications, including clinical diagnostics and drug discovery.

## 1. Introduction

Ventilator-associated pneumonia (VAP) stands out as the most prevalent hospital-acquired infection in intensive care units (ICUs), presenting significant challenges. VAP is associated with elevated mortality rates, prolonged antimicrobial usage, extended mechanical ventilation, and increased healthcare costs [1]. In developing nations, approximately 30% of patients on mechanical ventilation suffer from VAP, leading to substantial mortality rates ranging from 16% to 94% [2]. Histopathological examinations of VAP cases illustrate neutrophil infiltration, fibrin exudates, and cellular debris within intra-alveolar spaces, especially around terminal bronchioles, as a result of invasion by bacterial pathogens [3].

Currently, diagnosing VAP primarily relies on microbiological sampling of distal airways, though the distinction between lung parenchyma invasion and lower airway colonization remains contentious, and obtaining and processing these samples for preliminary or definitive results can be time consuming [4]. The electrochemical detection of pyocyanin presents a promising approach for Ventilator-Associated Pneumonia (VAP) diagnosis, offering potential advantages over existing diagnostic techniques. Electrochemical methods can provide rapid and sensitive detection of pyocyanin, a biomarker associated with Pseudomonas aeruginosa infection, which is a common cause of VAP. These methods offer quicker results compared to traditional microbiological cultures or polymerase chain reaction (PCR) approaches, enabling timely initiation of targeted antimicrobial therapy. Additionally, the potential for integration into portable point-of-care devices makes electrochemical detection user-friendly and suitable for bedside applications. While traditional methods may have proven sensitivity and specificity, the accessibility, ease of use, and speed of electrochemical detection could be advantageous for enhancing diagnostic efficiency, particularly in critical clinical settings where prompt and accurate diagnosis is paramount.

The absence of a consensus on early VAP diagnosis has led to subjective decision-making among medical teams, triggering debates when clinical suspicion of VAP arises based on various indicators. Inappropriate or delayed antibiotic use can escalate mortality rates, while excessive antibiotic use heightens morbidity, mortality, and bacterial resistance; thus, accurate and early VAP diagnosis is crucial to striking a balance between reducing mortality and preventing multi-drug resistance [4].

While chest radiography (CXR) is commonly employed for VAP diagnosis, its efficacy is limited. The gold standard is computed tomography (CT); however, it necessitates patient transportation, involves radiation exposure, and is costly. Point-of-care ultrasound, a non-invasive and non-irradiating bedside technique, has gained prominence in ICUs, aiding in the differential diagnosis of acute respiratory failure. Lung ultrasonography (LUS) can be integrated into clinically driven protocols to assist in this differentiation. However, diagnosing VAP in mechanically ventilated patients proves challenging due to possibilities such as non-infectious factors causing fever and pulmonary infiltration. Use of the clinical pulmonary infection score (CPIS) has been suggested for VAP diagnosis; however, it exhibits poor diagnostic performance, making it impractical for daily use. Even with enhancements such as direct gram-stain examination of tracheal aspirate and/or procalcitonin, previous studies indicate that the diagnostic accuracy of CPIS remains limited [5].

Pyocyanin, a virulence factor belonging to the phenazine group, is produced by *Pseudomonas aeruginosa* strains, enhancing their virulence by inducing the generation of reactive oxygen species such as hydrogen peroxide that disrupt various biological processes in host cells. Electrochemical techniques have made it possible to detect Pyocyanin due to their low detection limit, opening up opportunities for the development of Pyocyanin detection methods. Electrochemical sensors have gained popularity thanks to their affordability, ease of preparation, sensitivity, rapid response, and user-friendly operation, making them suitable for quick diagnosis and individual self-monitoring [6]. The key to effective nonenzymatic Pyocyanin detection lies in the design of the electrode surface, with a focus on synthesizing a catalyst with a porous structure. Micro/nanoporous morphologies offer numerous advantages, including abundant active sites, high permeability, and shorter charge transfer channels, making them highly attractive for sensor applications and ensuring enhanced Pyocyanin detection performance.

Graphene oxide ( GO ) has found extensive applications across diverse fields, including biomedicine, environmental applications, and agriculture, owing to its outstanding physical and chemical properties [7,8]. The hydrophilic reactive oxygen functional groups present on the surface of GO facilitate its dispersion in water [9]. Recent studies have highlighted the broad-spectrum bactericidal properties of GO against plant pathogenic bacteria and fungi [10]. Furthermore, the combination of GO with DNA-directed silver nanoparticles (Ag NPs) has proven to be effective in controlling bacterial spots on tomatoes [11]. Over the past decade, substantial research efforts have been dedicated to exploring the versatile applications of GO , a two-dimensional nanomaterial possessing advantages such as high electron mobility, chemical stability, ample surface area, and excellent thermal conductivity [12].

GO is a chemical derivative of graphene characterized by a theoretical surface area of approximately 736.6 m²/g and a zeta potential value of −113.77 mV, attributed to the presence of epoxide, carboxylic, and hydroxyl groups. These functional groups confer a negative charge and hydrophilicity, enabling easy dispersion of GO in aqueous solutions and forming stable suspensions [13]. This property renders GO an ideal host material for loading with nanoparticles such as copper oxide ( CuO ) and magnesium oxide ( MgO ). In this study, we introduce a nano-biocide based on GO−Cu−MgO , where copper–magnesium oxide nanoparticles ( Cu−MgO NPs) are immobilized onto GO sheets to form GO−Cu−MgO (GCM) NP composites. These composites exhibit strong Pyocyanin detection capabilities, and hold significant promise for a variety of applications.

This research explores the potential of GCM as a catalytic interface for enzyme-free Pyocyanin detection. A comprehensive set of characterization tests was performed to analyze the particle size, morphology, element composition, and surface structure of the prepared GO−Cu−MgO NP composites. Additionally, we propose that the GO−Cu−MgO NP composites can induce cell membrane damage, oxidative stress through reactive oxygen species (ROS), and DNA degradation. The improvement in electrocatalytic performance for efficient Pyocyanin detection is attributed to the synergistic effects of electrical interactions among the GCM components.

## 2. Materials and Experiments

### 2.1. Chemicals

Pyocyanin (CAS85-66-5/ref.10009594) was purchased from Cayman Chemicals, Estonia; 100 nmol ssDNA aptamer with sequence (5′- CTT CTG CCC GCC TCC TTC CTA GCC GGA TCG CGC TGG CCA GAT GAT ATA AAG GGT CAG CCC CCC AGG AGA CGA GAT AGG CGG ACA CT- 3′) was purchased from Integrated DNA Technologies, Belgium; and KMnO_4_, $H_{2}SO_{4}$ , $H_{2}O_{2}$ , HCl, ammonia water, $MgCl_{2}$ · $6H_{2}O$ , Nafion, ethanol, and graphite were acquired from Rankem, India. Cupric chloride (99%) was sourced from Nanjing Chemical Reagent Co., Ltd., Nanjing, China. Additionally, Sodium hydroxide (NaOH) (99%) was obtained from the same supplier. All of these raw materials were used directly without any additional purification steps. All materials were treated with distilled water throughout the preparation and characterization process. Furthermore, deionized (DI) water was used for the preparation of all reagents.

### 2.2. Synthesis of Nanocomposite

The modified Hummer method was employed to synthesize graphene oxide ( GO ) from graphite through oxidation. The synthesis of GO powder involved a series of steps. Initially, 1 g of graphite powder was mixed with 23 mL of concentrated $H_{2}SO_{4}$ (98%) while vigorously stirring in an ice bath for 30 min. Subsequently, 3 g of $KMnO_{4}$ was gradually added to the mixture with continuous stirring for 2 h while maintaining the temperature below 10 °C. The mixture was then heated to 35 °C and stirred continuously for another 2 h. Following this, 46 mL of distilled water was slowly added to the mixture while stirring for an additional 20 min. To stop the reaction, 140 mL of distilled water was added. The introduction of 30% $H_{2}O_{2}$ led to the bright yellow coloration of the mixture. The resulting sample was washed through repeated centrifugation with an HCl solution and distilled water. The sediment obtained from this process was dried under vacuum conditions, resulting in the formation of GO powder.

Cupric chloride underwent reduction and was then immobilized onto the surfaces of GO sheets with the assistance of ammonia water. The process began by dissolving 40 mg of GO powder in 40 mL of distilled water, forming a homogeneous GO suspension through 30 min of sonication. Next, 2 g of cupric chloride was added to the GO suspension and stirred for 30 min, then 1 mL of ammonia water was swiftly introduced into the mixture followed by vigorous stirring for 1 h. A specified amount of $MgCl_{2}\cdot 6H_{2}O$ (0.5 M) was stirred into 50 mL of the above solution on a hot plate. Throughout the stirring process at 80 °C for 4 h, the pH of the solution was maintained at 12 using NaOH (0.1 M). After stirring, the solution underwent centrifugation at 3500 rpm for 15 min. The resulting supernatant was collected and dried in an oven at 120 °C for 24 h. The collected powder was finely ground using a mortar and pestle, forming a fine powder termed graphene oxide–copper oxide-doped MgO (GCM).

### 2.3. Fabrication of Electrode

The catalytic ink was produced by subjecting 15 mg of the nanocomposite to sonication in an ice bath for 2 h. During this process, 20 μ L of Nafion and 1.5 mL of ethanol were added to the mixture. Nafion plays a crucial role in electrochemistry experiments by providing ionic conductivity, acting as an ion exchange membrane, preventing cross-contamination, aiding in electrode modification, enhancing stability and sensitivity, reducing interference, and facilitating gas diffusion, ultimately contributing to accurate and reliable electrochemical measurements. After sonication, 10 μ L of the resulting catalytic ink was deposited onto glassy carbon electrodes (GCE) with a diameter of 2 mm. The ink-coated electrodes were then left to dry at room temperature.

### 2.4. Characterization of Catalyst

The crystal phases were gathered utilizing an X-ray diffractometer (XRD-6100, LabX, SHIMADZU Ltd., Kyoto, Japan). FTIR (Fourier transform infrared) was performed for surface characterization of nanoparticles. Transmission Electron Microscopy (TEM) was employed to examine the morphology of the nanocomposite.

Furthermore, to dilute the aptamer, 95 μ L of molecular grade water (MGW) was added to 5 μ L of the aptamer, resulting in a final volume of 100 μ L. This created a stock solution with a concentration of 10 μ M. Subsequently, the working solution for the aptamer was prepared by mixing 10 μ L of the stock solution with 90 μ L of MGW, resulting in a final-concentration of 1 μ M. Similarly, for Pyocyanin, 50 mg of Pyocyanin was initially dissolved in 2 mL of MGW to create the stock solution. To prepare the working stock solution of 1 mg/mL, 40 μ L of the stock solution was mixed with 960 μ L of MGW. For the working solution with a concentration of 0.1667 mg/mL, 200 μ L of the 1 mg/mL Pyocyanin solution is mixed with 1 mL of MGW to mimic the environment of the throat trachea.

**Figure 1:**
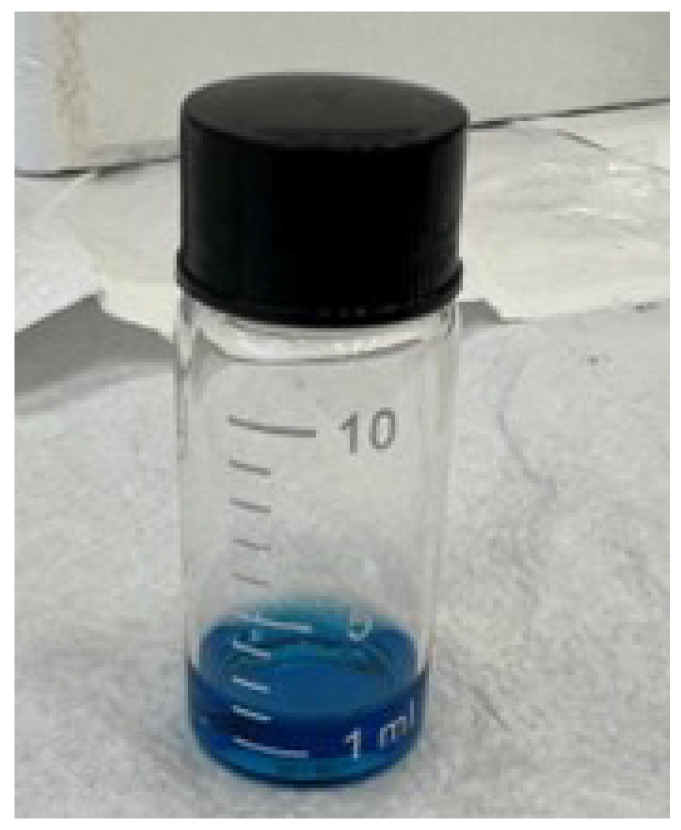
The prepared Pyocyanin working solution with a concentration of 0.1667 mg/mL, ready for downstream assays and experiments.

Figure 1 shows the resulting desired concentration of Pyocyanin for use in downstream assays and experiments.

## 3. Electrochemical Experiments

Multiple electrochemical experiments were performed using a Gamry Galvanostat (Ref 600), including cyclic voltammetry (CV), chronoamperometry, and electrochemical impedance spectroscopy (EIS). The setup involved a glassy carbon (GC) working electrode coated with catalytic ink, an Ag/AgCl reference electrode, and a graphite rod counter electrode. Pyocyanin detection was conducted in a 25 mL 0.5 M KOH electrolyte; the measurements were carried out under specific conditions, with the swept rates varying between 10 to 100 mVs^−1^.

## 4. Results and Discussion

### 4.1. Structural and Morphological Assessment

**Figure 2:**
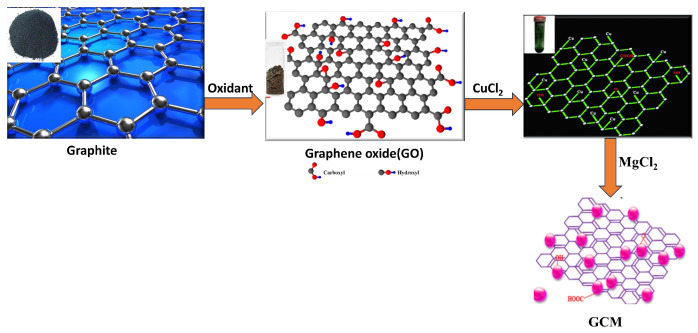
Detailed synthesis process of graphene oxide–copper oxide-doped MgO (GCM) fine powder.

**Figure 3:**
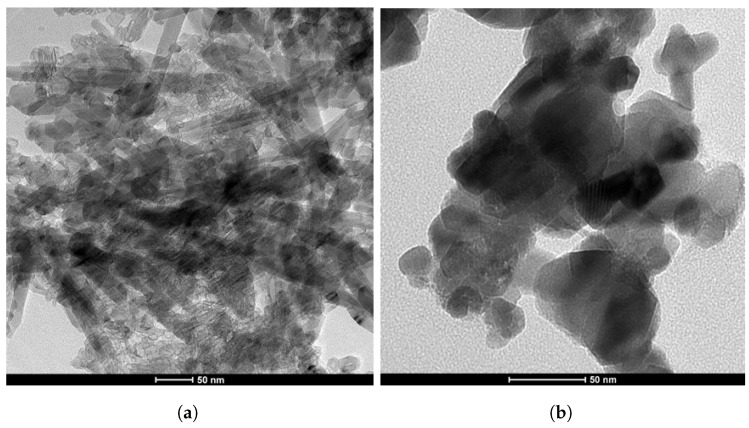
Transmission electron microscopy (TEM) images of two distinct composites: (**a**) GO composite and (**b**) GO-Cu-MgO composite.

In this study, copper (Cu) and magnesium (Mg) nanoparticles (NPs) were immobilized onto graphene oxide (GO) using ammonia as a reductant and stabilizer; the synthesis process is detailed in Figure 2. Transmission Electron Microscopy (TEM) was employed to examine the morphologies of the graphene oxide–copper and magnesium nanoparticle (GCM NP) composites. In Figure 3a, GO is depicted as a thin and semitransparent sheet; after the reaction, dark spots are observed immobilized on the GO sheets (Figure 3b). The lattice fringes of these dark spots on the GO correspond to the (111) lattice plane of the copper nanoparticles (Cu NPs), with a measured size of approximately 31 nm [14]. Previous results suggest that these small particle sizes could enhance antibacterial properties due to increased opportunities for interaction with bacteria [15]. The TEM analysis in Figure 3 confirms the successful formation of the nanocomposite, revealing various regions with different thicknesses of GO platelets and exhibiting a sheet-like morphology with varying degrees of transparency. The dark areas represent thickly stacked nanostructures with multiple layers of graphene oxide and/or graphene along with some oxygen functional groups, while the transparent regions indicate thinner films comprising only a few layers of graphene oxide, resulting from the exfoliation of the stacked nanostructure.

**Figure 4:**
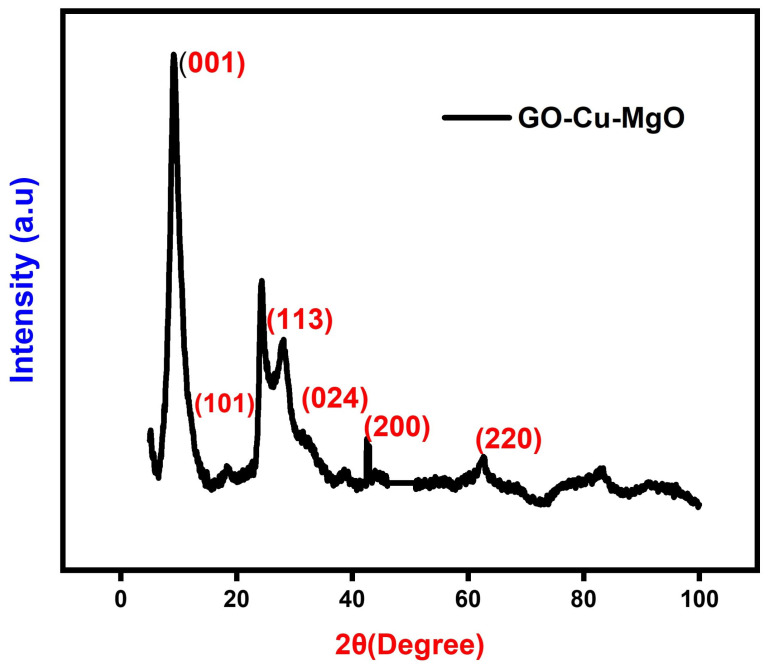
XRD pattern of GCM.

The X-ray diffraction (XRD) patterns depicted in Figure 4 offer further insights into the microstructures of the GCM nanoparticles (NPs). A prominent peak in the XRD pattern at $2\theta =10.43^{\circ }$ is consistent with the crystal structure of graphene oxide ( GO ) [16]. However, this peak is absent in the XRD pattern of GO−Cu NPs, indicating that the presence of copper nanoparticles (Cu NPs) altered the surface structure of GO . In the XRD pattern of GO−CuO NPs, diffraction peaks observed at $16.22^{\circ }$ , $32.26^{\circ }$ , and $39.68^{\circ }$ were identified as the (101), (113), and (024) crystalline planes, respectively, resembling the crystal structure of paratacamite ( $Cu_{2}{\left(OH\right)}_{3}Cl$ , JCPDS file no. 87-0679). The peaks at $42.5^{\circ }$ and $62.3^{\circ }$ correspond to (200) and (220) planes, respectively, confirming that magnesium oxide ( MgO ) exhibits a cubic structure consistent with JCPDS 75-1525 [17]. The doping process exhibited identical diffraction peaks, indicating that a minimal amount of GO dopant content remained undetectable. The average crystallite size was calculated using the Debye–Scherrer equation, yielding a value of 23.28 nm. Additionally, the calculated d-spacing of 0.31 nm was attributed to the (200) lattice plane of cubic MgO .

**Figure 5:**
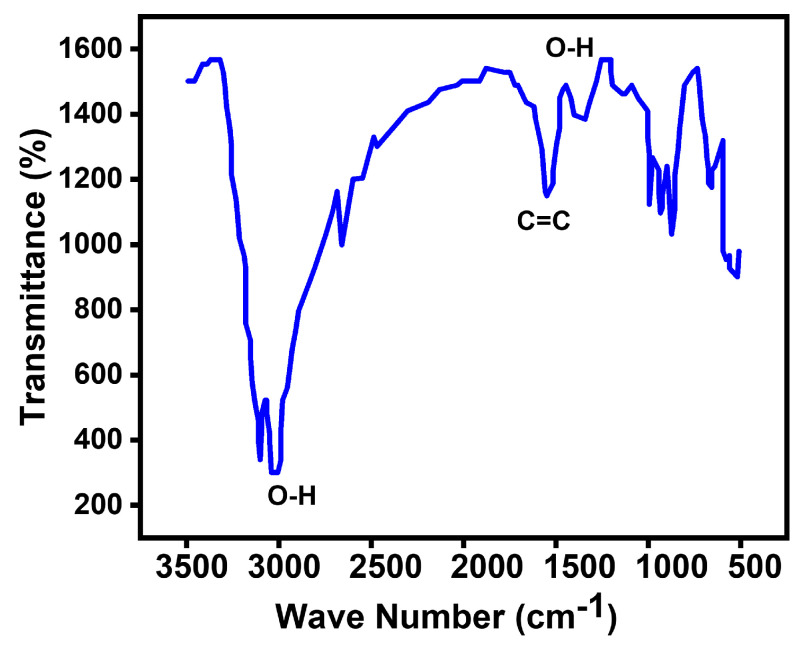
FTIR spectra of GCM.

In this study, alterations in the functional groups were investigated using FTIR spectroscopy. The FTIR results displayed distinct peaks corresponding to various functional groups in the spectrum of graphene oxide ( GO ). These peaks included stretching vibration peaks of O−H (3427 cm^−1^), C−C (1655 cm^−1^), and C−O (1286 cm^−1^). Additionally, asymmetric stretching vibrations of C−H bonds in $-CH_{3}$ and $-CH_{2}$ (2924 cm^−1^) and in-plane bending vibrations of C−H (1425 cm^−1^) were observed (Figure 5).

Upon the reaction between GO and copper nanoparticles ( Cu NPs), significant changes in the functional groups were detected. The strong peak at 3427 cm^−1^ split into two adsorptions of O−H stretching vibrations at 3446 cm^−1^ and 3354 cm^−1^. Furthermore, the absorption band of C−C at 1655 cm^−1^ shifted to 1624 cm^−1^. Notably, the absorption bands at 2924 cm^−1^, 1425 cm^−1^, and 1286 cm^−1^ disappeared. Conversely, new peaks emerged, including the O−H stretching vibration at 1385 cm^−1^, the C−H out-of-plane bending vibrations at 987, 920, and 847 cm^−1^, and the Cu−O stretching vibrations in monoclinic copper oxide below 600 cm^−1^ [17,18]. These changes in the functional groups strongly suggest the successful immobilization of copper nanoparticles on the surface of the graphene oxide.

The observed transmittance peak at around 1699 cm^−1^ was attributed to the characteristic stretching vibration of hydroxyl groups (alcohol) resulting from the reaction between the surface of magnesium oxide ( MgO ) and water vapors in the air [18]. Upon the addition of graphene oxide ( GO ), a reduction in peak intensity was observed, likely due to the presence of dopant sheets enveloping the MgO . The band at 1450 cm^−1^ was linked to the asymmetric stretching vibrations of carbonate ions ( C−O ), with corresponding bending vibration peaks noted around 865 and 867 cm^−1^. However, a decrease in the intensity of these bands was observed for the GO−CuO -doped MgO sample. Furthermore, a band around 1443 cm^−1^ indicated the presence of a magnesium–oxygen ( Mg−O )-characterized stretching vibration [19,20,21].

### 4.2. Electrochemical Profile for Detection of Pyocyanin

**Figure 6:**
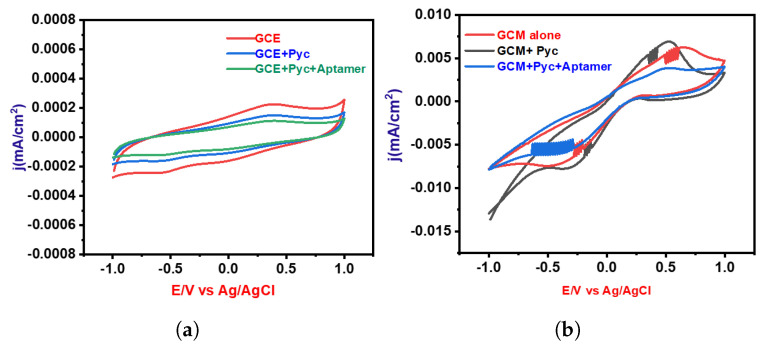
Cyclic voltammetry (CV) analyses for two electrode configurations: (**a**) Blank Glassy Carbon Electrode (GCE) and (**b**) electrode coated with Graphene Oxide–Copper–Magnesium Oxide (GO-Cu-MgO).

The electrochemical profile for Pyocyanin detection was systematically investigated through cyclic voltammetry (CV) experiments, primarily utilizing a Glassy Carbon Electrode (GCE). The preliminary phase of experimentation involved two distinct conditions: one devoid of Pyocyanin and the other with the presence of Pyocyanin, as illustrated in Figure 6a. These initial experiments indicated that the GCE alone exhibited limited efficacy in detecting Pyocyanin. In response to this observation, a subsequent series of CV analyses were conducted using a GCE coated with a specialized Graphene–Carbon Nanotube Composite (GCM) material. This coating was strategically designed to augment the detection capabilities of the GCE. The analysis was carried out in three distinctive cases, each serving a unique purpose directed towards comprehensively understanding the electrochemical behavior. The first case involved performing CV without Pyocyanin in order to establish a baseline for the electrode’s behavior. This baseline provided crucial insights into the intrinsic characteristics of the coated electrode in the absence of the target analyte. The second case entailed the introduction of Pyocyanin, facilitating an assessment of the electrode’s response to the presence of this specific analyte. This step was essential for understanding the electrode’s sensitivity and selectivity towards Pyocyanin. Finally, the third case integrated both the aptamer and Pyocyanin into the experimental setup. This configuration allowed for a thorough evaluation of the synergistic effect between the aptamer and Pyocyanin on the electrode’s performance. The results of these experiments are depicted in Figure 6b, illustrating the variations in the electrochemical response under different conditions.

This manuscript explores the integration of aptamers, unique biomolecules with high specificity and affinity, into the field of diagnostic assays, specifically focusing on the detection of pyocyanin, a critical parameter in certain diagnostic scenarios. Aptamers, whether composed of short single-stranded DNA or RNA, serve as precise molecular tools designed to bind specifically to target molecules. In the context of Pyocyanin detection, aptamers act as molecular recognition elements, enabling the development of highly sensitive and specific diagnostic assays. The versatility of aptamers is highlighted by their ability to be seamlessly integrated into biosensor platforms, where they serve as the biological recognition component. Such integration significantly improves the sensitivity and specificity of biosensors, allowing for accurate and reliable measurements of Pyocyanin levels and ultimately positioning aptamers as promising tools for point-of-care diagnostics.

This manuscript delves into the unique properties of aptamers, emphasizing their ability to form supramolecular multicomponent structures through hybridization. This feature enhances the versatility of aptamers, making them valuable for creating complex molecular architectures. One notable application of aptamers is their potential to serve as carriers for therapeutic agents, including small interfering RNA (siRNA). Due to their specific binding to cell-surface targets, aptamers can be employed for targeted delivery of therapeutic payloads, improving precision and minimizing off-target effects. In the specific context of Pyocyanin detection, this manuscript explores the use of aptamers to develop a method called GCM and demonstrates its effectiveness in detecting Pyocyanin. Our study reveals that the presence of Pyocyanin induces a distinct response in the GCM device, likely manifested as a higher current, which is attributable to the specific binding of Pyocyanin molecules to the immobilized aptamers on the GCM sensor. This molecular interaction results in measurable changes in electrical properties, offering a clear indication of the presence of Pyocyanin. Importantly, our research underscores the need to comprehend molecular interactions and the impact of aptamers on the detection process. The integration of aptamers into specific detection techniques such as GCM is emerging as a powerful approach for the sensitive and specific detection of target molecules such as Pyocyanin, showcasing the potential of aptamers in biosensing and diagnostic applications. The utilization of Cu−Mg doped graphene oxide enhances Pyocyanin sensing through a synergistic effect that optimizes the material’s electrical and catalytic properties. First, the incorporation of copper (Cu) and magnesium (Mg) into the graphene oxide structure introduces additional active sites for Pyocyanin binding. The presence of these dopants enhances the material’s affinity towards Pyocyanin molecules, promoting a more effective and selective sensing capability. Second, the doped graphene oxide exhibits improved electrical conductivity compared to pristine graphene oxide. This enhanced conductivity facilitates efficient electron transfer during the electrochemical sensing process. As Pyocyanin undergoes redox reactions, the modified graphene oxide’s superior electrical conductivity ensures rapid and sensitive detection even at low concentrations.

**Figure 7:**
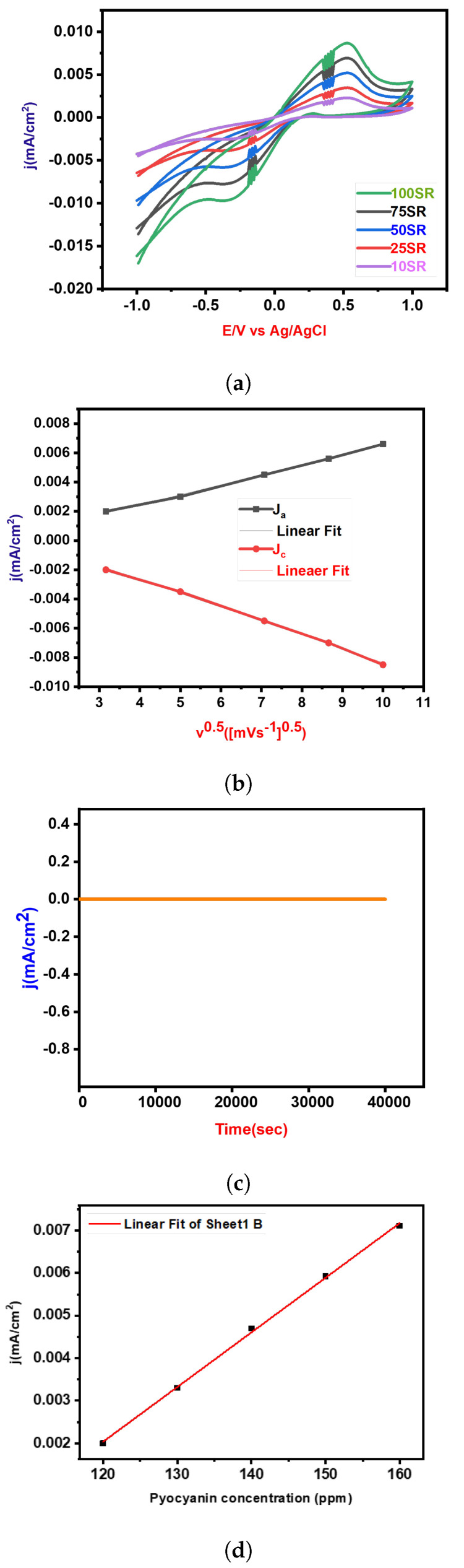
Complete analysis: (**a**) Cyclic Voltammetry (CV) curves illustrating electrochemical behavior at various scan rates (mV/s); (**b**) peak current density (mA/cm²) corresponding to anodic and cathodic peaks plotted against the square root of scan rate (mV/s); (**c**) durability assessments for Pyocyanin detection, demonstrating the long-term stability and repeatability of the electrochemical sensor; (**d**) effect of Pyocyanin concentration.

In Figure 7a, the cyclic voltammetry (CV) measurements for Pyocyanin detection using GCM in a KOH solution at neutral pH are presented. The scan rate ranged from 10 to 100 mV/s. A notable increment in redox peak current density was observed during the reduction process, as depicted in Figure 7b. This suggests an enhanced electrochemical response with increasing scan rates (SR). Furthermore, Figure 7b displays the relationship between the square root of the scan rate and the corresponding peak current densities of the GCM electrode. This relationship sheds light on the electrochemical behavior and performance of the GCM-modified electrode with varying scan rates, providing valuable insights into its kinetics and efficiency in Pyocyanin detection. To further understand the analytical capabilities of the GCM-modified electrode, linear regression analysis was performed for the redox peak current in Pyocyanin detection. The details of this analysis are comprehensively outlined in Table 1, providing key parameters that characterize the electrochemical response and detection efficiency of Pyocyanin using the GCM-modified electrode. In addition to assessing detection efficiency, durability assessments were carried out to evaluate the long-term stability and reliability of GCM in detecting Pyocyanin. Chronoamperometry was conducted over a duration of 10 h and at a potential of 0.3 V. Remarkably, as depicted in Figure 7c, the results highlight that the GCM-modified electrode exhibited significantly higher current retention even after the 10 h duration. This observation indicates superior stability compared to other electrodes, underlining the potential of GCM for robust and enduring Pyocyanin detection applications. Cyclic Voltammetry (CV) was employed as a diagnostic tool to investigate the electrochemical response of the electrocatalyst in detecting the Pyocyanin concentration. As illustrated in Figure 7d, the CV curve presents the electrochemical behavior across various Pyocyanin concentrations from 120 to 160 ppm. The experiments were conducted using a scan rate of 50 mV s^−1^ and within a potential range from −0.1 V to 1 V. The distinctive feature in Figure 7d is the observable enhancement in the oxidation/reduction peak current with increasing concentrations of Pyocyanin in the electrolyte. This trend signifies a direct correlation between the concentration of Pyocyanin and the electrochemical response of the system. Notably, the current density experienced a significant surge as the Pyocyanin concentration transitioned from 120 to 160 ppm, escalating from 0.002 to 0.007 mA/cm^2^. This observation indicates a concentration-dependent behavior, with higher concentrations of pyocyanin concentration resulting in an intensified electrochemical response. The increase in current density implies more pronounced electrocatalytic activity at elevated Pyocyanin concentrations, providing valuable insights into the sensitivity and detection capabilities of the electrocatalyst. The limit of detection (LOD) was evaluated using the 3 σ /m, where σ is the standard deviation of the intercept and m represents the slope of the calibration plot (R-Square (COD)=0.99902;  y=1.28338E−4x+(−0.01336±3.26589E−4)) . Electrochemical assessments conducted on the fabricated electrode demonstrated impressive sensitivity to the Pyocyanin concentration, as evidenced by an exceptional detection limit of 43 ppm.

**Figure 8:**
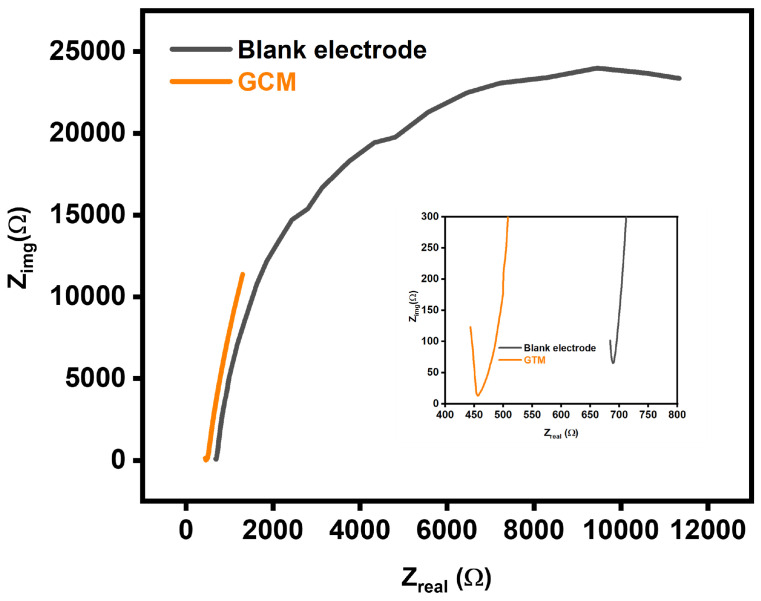
Electrochemical Impedance Spectroscopy (EIS) spectra comparing the impedance characteristics of the Glassy Carbon Modified (GCM) electrode and blank electrode, providing insights into their electrochemical behavior and surface properties.

In addition to cyclic voltammetry (CV), electrochemical impedance spectroscopy (EIS) was employed in this study to further elucidate the electrochemical properties of the Glassy Carbon Electrode (GCE) and its modification with a Graphene Carbon Matrix (GCM). The EIS experiments were conducted at a fixed potential of 1 V vs. Ag/AgCl, spanning a frequency range from 0.1 Hz to 100 kHz. Analysis of the impedance data revealed the charge transfer resistance (Rct) for both the unmodified (blank) electrode and the GCM-modified electrode. The Rct for the blank electrode was determined to be 695, indicating resistance to charge transfer at the electrode interface. In contrast, the GCM-modified electrode exhibited a significantly lower Rct value of 457. This substantial reduction in charge transfer resistance is a pivotal observation, suggesting an improvement in the electrochemical properties of the GCM-modified electrode. The inset in the graph focused on the linear portion of the impedance curve, highlighting that the composite electrode displayed notably lower resistance compared to the blank electrode, especially at lower frequencies. This reduction in resistance implies enhanced mass transportation, allowing for more efficient electron conduction pathways and expanding the reactive surfaces on the GCM-modified electrode. The lower charge transfer resistance observed in the GCM electrode is a critical indicator of improved electrochemical performance. This enhancement is further illustrated in Figure 8, which visually represents the enhanced diffusion of the electrolyte on the electrode surface, supporting the idea of improved mass transportation. As indicated by the lower Rct, the increased reactive surfaces on the GCM electrode contribute to its effectiveness in facilitating efficient electrolyte diffusion and electron conduction. The selection of a fixed potential of 1 V vs. Ag/AgCl in the electrochemical impedance spectroscopy (EIS) study was likely a strategic choice aimed at optimizing conditions for investigating charge transfer resistance (Rct) and other electrochemical properties. This voltage setting ensures a sufficient driving force for the electrochemical reactions under study while avoiding potential-related issues such as unwanted side reactions and electrode damage. The determination of Rct values for both the unmodified (blank) electrode and the Graphene Carbon Matrix (GCM)-modified electrode was carried out by analyzing the impedance spectra over a sweeping frequency range from 0.1 Hz to 100 kHz. In EIS analysis, Nyquist plots are commonly employed to interpret impedance data by presenting it in the complex plane, with the real part (Zreal) on the x-axis and the imaginary part (Zimag) on the y-axis. The semicircular portion of a Nyquist plot typically represents the charge transfer process. It is noteworthy that shifts along the Zreal axis may not necessarily correspond to changes in Rct values, suggesting that alterations in Zreal are not solely indicative of changes in charge transfer resistance. The lower frequency range, corresponding to high Zreal, indicates that the charge transfer resistance is more dominant at lower frequencies. The observed decrease in charge transfer resistance in the GCM-modified electrode in comparison to the blank electrode (695 for blank and 457 for GCM-modified electrode) is a significant finding. This reduction implies improved electrochemical properties with the GCM modification, indicating a decrease in hindrance to charge transfer. This is crucial for facilitating efficient electrolyte diffusion and electron conduction, leading to improved electrochemical performance. The inset in the graph, focusing on the linear portion of the impedance curve, underscores the reduction in resistance, particularly at lower frequencies.

**Figure 9:**
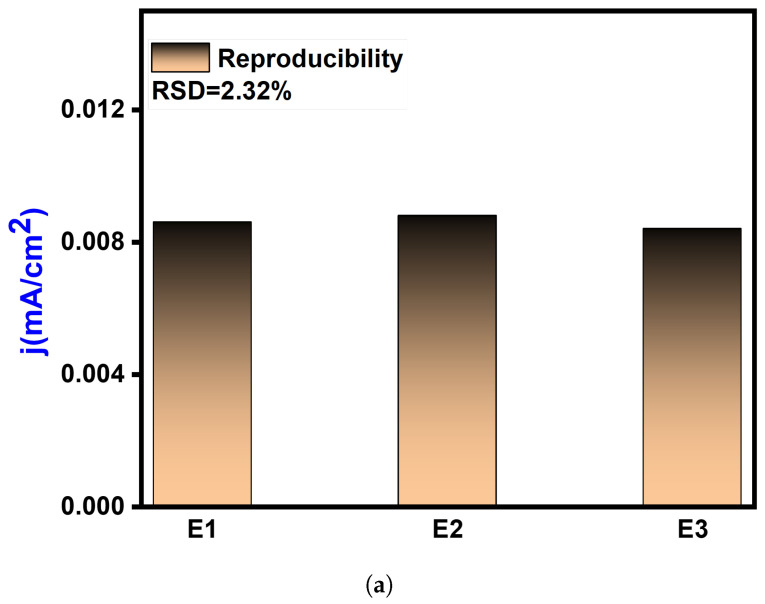

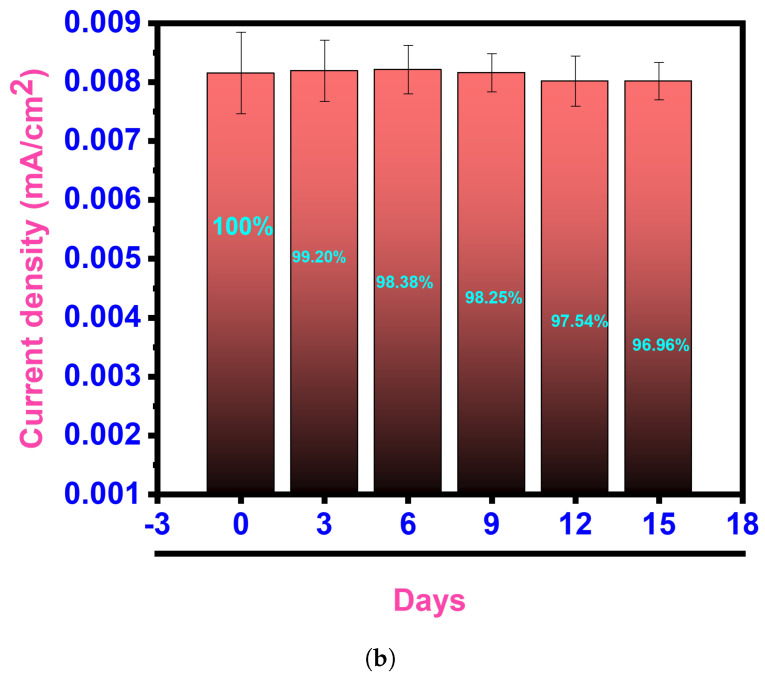
Reproducibility and stability analysis: (**a**) reproducibility assessment of the electrochemical sensor, demonstrating consistent and repeatable results under controlled conditions and (**b**) electrochemical sensor response after the introduction of Pyocyanin, showcasing the stability and performance of the sensor upon analyte addition and validating its sensitivity and specificity.

When assessing sensing devices, it is crucial to consider their reproducibility, repeatability, and stability as significant characteristics. Repeatability refers to the consistency of successive readings obtained with the same electrode. To verify the repeatability, a single modified GCE was utilized thrice daily. The relative standard deviation (RSD) of the repeatability was calculated to be 1.03%, indicating that the proposed sensor exhibits exceptional repeatability. Reproducibility refers to the similarity of results obtained using multiple instances of the same modified electrodes and measurement techniques. In this study, after introducing Pyocyanin, the CV responses of three independent electrodes were recorded under ideal conditions. The RSD of the response currents for different GCM NC electrodes was only 2.32%, indicating high consistency (Figure 9a). The durability of the suggested electrochemical sensor was assessed by storing the GCM NC-modified GCE electrodes in the air and monitoring the current response. As depicted in Figure 9b, after 15 days in the air the response current of the GCM NC-modified GCEs retained around 96.96% of its initial value, demonstrating good storage stability. Furthermore, the sensor’s reactivity towards the substrate takes some time to reach a steady state from the moment it is immersed in the electrolyte. It was observed that it took 2 s for the sample to achieve a steady current after being added to the sensor.

## 5. Conclusions

The exploration and optimization of catalyst composition, morphology, and surface modifications represent essential steps in advancing electrochemical detection methods for Pyocyanin. Our findings suggest that a GCM catalyst without an aptamer can provide a rapid and cost effective diagnostic tool for VAP casued by *Pseudomonas aeruginosa*. This electrochemical sensor has the potential to be made disposable for in vivo applications. Using a GCM catalyst offers synergistic effects that enhance the overall electrochemical performance of the detection process along with its sensitivity and selectivity. In conclusion, using GCM for electrochemical detection shows great promise in achieving sensitive and selective Pyocyanin detection across different sample types. The GCM catalyst provides a conductive and catalytically active surface that facilitates the electrochemical oxidation and reduction of Pyocyanin, resulting in measurable changes in electrical current that allow for determination of the Pyocyanin concentration in the sample solution. Incorporating GCM into the catalyst results in synergistic effects that improve the overall electrochemical performance of the detection process. Furthermore, the catalyst can be modified or functionalized to further enhance its Pyocyanin detection capabilities. In all, the electrochemical detection of Pyocyanin using a GCM catalyst holds tremendous potential for a wide range of applications.

## Figures and Tables

**Table 1:** Linear regression details of the redox peak current for detecting Pyocyanin.

Plot	Anodic	Cathodic
**Weight**	No Weighting	
**Intercept**	−2.64399E−4±1.47431E−4	0.00113±1.91124E−4
**Slope**	6.79243E−4±2.0445E−5	−9.48961E−4±2.65041E−5
**Residual Sum of Squares**	3.79268E−8	6.37382E−8
**Pearson’s r**	0.99864	−0.99883
**R-Square (COD)**	0.99729	0.99767
**Adj R-Square**	0.99639	0.99689

## Data Availability

The data will be available upon to request.
